# Why call bodily sense making “languaging”?

**DOI:** 10.3389/fpsyg.2014.01286

**Published:** 2014-11-07

**Authors:** Giovanna Colombetti

**Affiliations:** Department of Sociology, Philosophy and Anthropology, University of ExeterExeter, UK

**Keywords:** primary intersubjectivity, sense making, languaging, affectivity, language

I am sympathetic to Jensen's aim to “bring language and emotion back together.” To speak is among other things to communicate one's affective state to others, and this communication is typically effectuated by embodied agents whose affective state also manifests in their face, posture, gestures, facial expressions, and tone of voice. When I talk to someone I usually look at them in a more or less engaged way; I may also smile or frown, nod sympathetically or shake my head in disapproval, giggle, laugh, gesticulate, alter the volume and pace of my voice, and so on. These actions are partly responses to what to the other says and how he says it, and often have the function of affecting how the interaction continues (a nod may communicate approval at what is being said as well as encouragement to carry on). The interactions analyzed by Jensen nicely illustrate clear instances in which language is continuous and integrated with other types of bodily engagement with other people. In addition to being responses to others, my actions, when I speak, are also often related to the meaning of my words. As I am telling my friend about the climb I did on the weekend, I move my head down and close my eyes when I tell her how scared I was of the height and that I did not want to look down; I reproduce climbing movements with my hands or even the rest of the body when I tell her about a difficult passage; I spread my arms when I tell her about the 360-degree view from the top of the mountain, etc. Here as well we can see a continuity between language in the sense of well-formed word-based speech, and a variety of communicative bodily gestures.

So, I agree with much of what Jensen says in his article. However, I remain unclear about his use of the notion of “languaging,” particularly about its relationship to bodily sense making. I understand the point of talking of “languaging” to denote “language as an activity” (p. 2),^1^ namely as a process and as a behavior rather than as a static system of symbols and rules. But the notion of languaging in Jensen's paper *also* appears to be stretched to include *all* instances of bodily sense making, which I think is problematic. For example, at the beginning of the article Jensen writes that languaging is first-order “behavior or whole-body sense making” (p. 1, footnote 1); and at the end he suggests that preparing a meal in the presence of others, yet without using words, can also be seen as an instance of languaging—if done “in a very distinct way” (p. 12) that communicates some kind of affect (“a hectic, hasty, and perhaps even angry way of cooking,” p. 12).

Now, “whole-body sense making” may well be what grounds word-based linguistic phenomena, but it can also occur *before* language is acquired—so it's not clear that we should call all cases of bodily intersubjective sense making “languaging.” We know from developmental psychology that already shortly after birth infants interact with their caregivers by responding to bodily contact, vocalizations, and gaze direction (e.g., Tronick et al., 1979; Tronick, 2003). In the first year of life, infants engage in progressively richer interactions with the caregiver, in what is known as “affect attunement,” i.e., the cross-modal matching of vocalizations and bodily movements in terms of rhythm and intensity (e.g., Stern, 1985; Legerstee et al., 2007). The term “primary subjectivity” (Trevarthen, 1979), which Jensen mentions, refers to these and other skills that are present very early in development—such as imitation, a capacity to distinguish between inanimate objects and people, and a responsiveness to others' facial expressions. These skills arguably embody a pragmatic form of understanding others (e.g., Gallagher, 2001), also dubbed a “participatory sense making” (De Jaegher and Di Paolo, 2007). Although these forms of bodily attunement do not disappear once language is acquired, and may be necessary for language acquisition (including systematicity and compositionality), in infants they seem to be best characterized as *pre*linguistic, as they do not require the capacity to utter words and meaningful sentences. Thus, to characterize them as instances of languaging, where “languaging” is (also) taken to denote “language as an activity,” seems misleading. Moreover, to do so may even convey the message that forms of intersubjective bodily attunement are immature forms of sense making, waiting to be fully realized once language is acquired, rather than complete and autonomous stages of development. Incidentally, I do not think that Jensen believes this is the case, given that at some point he writes that “the contours of languaging, in its most basic form, are definitely grounded in such early intersubjective behaviors” (p. 6)—namely, he seems to think that not all instances of bodily intersubjectivity are forms of languaging. But then we are left with the question of what distinguishes the two.

I thus agree with Jensen when he acknowledges, at the end of his article, that his approach raises serious conceptual challenges. As *conceptual*, however, they will not be answered by performing “many further studies” (p. 12), but only by clarifying one's theoretical framework and adopting a consistent terminology.

## Conflict of interest statement

The author declares that the research was conducted in the absence of any commercial or financial relationships that could be construed as a potential conflict of interest.

## Footnotes

^1^Page numbers refer to the online version of Jensen's article.
